# Can diet niche partitioning enhance sexual dimorphism?

**DOI:** 10.1002/ece3.9599

**Published:** 2022-12-18

**Authors:** Joshua T. Bauld, Katharine A. Abernethy, Jason Newton, David Lehmann, Isabel L. Jones, Luc F. Bussière

**Affiliations:** ^1^ Biological and Environmental Sciences University of Stirling Stirling UK; ^2^ Institut de Recherche en Ecologie Tropicale CENAREST Libreville Gabon; ^3^ National Environmental Isotope Facility Scottish Universities Environmental Research Centre East Kilbride UK; ^4^ Agence Nationale des Parcs Nationaux (ANPN) Libreville Gabon; ^5^ Biology and Environmental Sciences and Gothenburg Global Biodiversity Centre University of Gothenburg Gothenburg Sweden

**Keywords:** disruptive selection, ecological character displacement, evolution, resource competition, sexual dimorphism, sexual selection

## Abstract

Classic evolutionary theory suggests that sexual dimorphism evolves primarily via sexual and fecundity selection. However, theory and evidence are beginning to accumulate suggesting that resource competition can drive the evolution of sexual dimorphism, via ecological character displacement between sexes. A key prediction of this hypothesis is that the extent of ecological divergence between sexes will be associated with the extent of sexual dimorphism. As the stable isotope ratios of animal tissues provide a quantitative measure of various aspects of ecology, we carried out a meta‐analysis examining associations between the extent of isotopic divergence between sexes and the extent of body size dimorphism. Our models demonstrate that large amounts of between‐study variation in isotopic (ecological) divergence between sexes is nonrandom and may be associated with the traits of study subjects. We, therefore, completed meta‐regressions to examine whether the extent of isotopic divergence between sexes is associated with the extent of sexual size dimorphism. We found modest but significantly positive associations across species between size dimorphism and ecological differences between sexes, that increased in strength when the ecological opportunity for dietary divergence between sexes was greatest. Our results, therefore, provide further evidence that ecologically mediated selection, not directly related to reproduction, can contribute to the evolution of sexual dimorphism.

## Graphical abstract

Theoretical and empirical work suggests that between‐sex competition for resources plays a role in the evolution of sexual dimorphism. We investigated this concept by analyzing cross‐species relationships between the extent of sexual dimorphism and published sex differences in tissue stable isotope values, which are a proxy of dietary differences. We found that sexual dimorphism is related to sex differences in trophic level, in directions and contexts consistent with those expected if resource competition is involved in the evolution of sexual dimorphism.

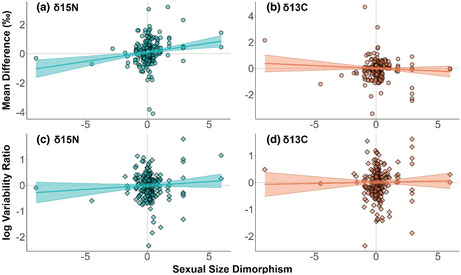

## 1 INTRODUCTION

### 1.1 Sexual dimorphism

Classic evolutionary theory posits that anisogamy, or variation between males and females in gametic investment, causes sex differences in optimum life history and reproductive strategy (Andersson, 1994; Lehtonen et al., 2016). Evolution toward these sex‐specific optima ultimately produces phenotypic differences between males and females, known as sexual dimorphism (Parker & Pizzari, 2015). One frequently observed sex difference is sexual size dimorphism in which the mean body size of one sex exceeds that of the other (Fairbairn et al., 2007). Size dimorphism is typically predicted to arise via sexual selection when the reproductive success of one sex is limited by mating opportunities, and large size allows individuals of that sex to increase their matings. For example, the biggest male southern elephant seals (*Mirounga angustirostris*) are the best able to monopolize females at breeding colonies (Le Boeuf et al., 2019). Alternatively, or additionally, size dimorphism may be favored by fecundity selection, when one sex's reproductive success is limited by gamete production and gamete production relates to body size, such as in emydid turtles (Emydidae), in which larger females produce more eggs and are therefore more fecund (Stephens & Wiens, 2009). It is this traditional view that size dimorphism is primarily attributable to reproductive differences, which is best evidenced and dominates scientific literature and consensus (Blanckenhorn, 2005).

However, as far back as Darwin's discussions of their “habits of life” (Darwin, 1871, p.254), authors have noted that sexual dimorphisms can relate not only to each sex's reproductive success but also to their respective ecologies (Shine & Goiran, 2021; Temeles et al., 2000; Wasiljew et al., 2021). Divergence of the sexes along various biotic and abiotic niche axes (e.g., temperature, diet, habitat) should facilitate intersex niche partitioning, which may covary with sexual dimorphism in traits relevant to ecology (Butler et al., 2007; Herrel et al., 1999). For example, in the seabird *Sula nebouxii*, larger females dive deeper and feed on larger prey than their male counterparts (Zavalaga et al., 2007).

Such ecological sexual dimorphisms could arise as an indirect, ecological consequence of dimorphism due to reproductive differences, or directly via ecological causation. An example of ecological dimorphism arising as an apparent consequence of reproductive differences can be found in mouthbrooding cichlid fishes. In species in which only one sex carries out mouthbrooding, sex differences in diet also arise as a consequence of distinct reproductive roles, as the mouthparts involved also influence foraging (Ronco et al., 2019). Ecological sexual dimorphisms arising as a consequence of reproductive differences are often considered the most parsimonious explanation for their existence (De Lisle, 2019; Shine, 1989).

### 1.2 Ecological character displacement between sexes

Theoretical work also suggests, however, that ecological sexual dimorphisms can arise solely from ecologically mediated natural selection if frequency‐dependent competition for a resource produces disruptive selection. Under these circumstances, the sexes could evolve toward distinct phenotypes, which maximize resource acquisition, by facilitating divergence along niche axes (Slatkin, 1984). This process of ecological character displacement between sexes is analogous to that between nascent species and offers an alternative evolutionary outcome to frequency‐dependent resource competition (Bolnick & Doebeli, 2003).

One issue with theoretical models of ecological character displacement, however, is the lack of an a priori justification for character displacement between sexes, as opposed to random subsets of populations (Bolnick & Doebeli, 2003). As a result, an integrated view of reproductive competition and ecological character displacement provides the best model for the evolution of ecological sexual dimorphisms (De Lisle, 2019, 2021). From this perspective, initial phenotypic divergence and/or differing ecological optima between the sexes may usually arise due to anisogamy and reproductive differences (Maklakov et al., 2008; Schärer et al., 2012). For instance, reproductive differences may create sex differences in nutritional optima, leading to divergent foraging decisions and resource allocation (Morehouse et al., 2020; Raubenheimer & Simpson, 2018). These initial differences may then be acted upon by disruptive natural selection, such that the observed differences between sexes emerge through both sexual and ecologically mediated selection. For example, intra‐specific reversals in the direction of python (*Morelia spilota*) size dimorphism track interpopulation differences in mating systems, but the degree of size dimorphism relates to available prey sizes (Pearson et al., 2002).

The ecological character displacement hypothesis for sexual dimorphism is perhaps best evidenced by a series of experiments by De Lisle and Rowe (2015) in which male and female salamanders (*Notophthalmus viridescens*) were placed in semi‐natural mesocosms, at various competitor densities. In this species, sexual dimorphism in body size and feeding morphology corresponds to sex differences in diet and microhabitat; meaning females may compete more strongly with other females and males with other males. In De Lisle and Rowe's study, growth rates were lower in high‐density mesocosms, suggesting that competition impacts fitness for which growth rate is a proxy. Furthermore, females grew faster in mesocosms with a male‐biased sex ratio. Females, therefore, had higher fitness when alongside a greater proportion of male competitors, with which they should compete for less, suggesting that sexual dimorphism somewhat reduced competition. Given such evidence, it is conceivable that ecologically mediated selection can play a role in the evolution of ecological sexual dimorphisms.

### 1.3 How important is ecological character displacement?

An outstanding and unresolved question concerns the importance, across taxa, of ecologically mediated selection for creating diversity in sexual dimorphisms. Here we address this question by investigating general associations, across species, between size dimorphism, and ecological divergence in trophic level and basal carbon resources. A central tenet of the ecological character displacement hypothesis is that the degree of dimorphism should scale positively with the degree of ecological divergence between sexes (De Lisle & Rowe, 2015). It, therefore, follows that more sexually dimorphic species should generally show greater ecological divergence than less dimorphic species. Of course, associations between dimorphism and ecological sex differences would not necessarily indicate causation because dimorphism in any individual species may have arisen via ecological causation or as an indirect consequence of reproductive differences. Associations would, however, be consistent with predicted outcomes of ecological character displacement working in isolation or reproductive differences creating the *opportunity* for disruptive ecologically mediated selection and would suggest a stronger relationship between sexual dimorphism and ecology than previously appreciated.

Ecological divergence related to size dimorphism could be exhibited by sex differences in ecological mean if, for example, dimorphism impacts the prey available to each sex, leading them to feed, on average, at different trophic levels (Mills et al., 2021). Furthermore, sexual size dimorphism could lead to sex differences in ecological variation via numerous mechanisms. For example, the larger sex may be more variable if large size confers access to a greater range of resources (Voigt et al., 2018) or the smaller sex more variable if they are competitively subordinate (Wan et al., 2013). We therefore examine associations between size dimorphism and ecological differences in foraging between males and females, measured using stable isotope analysis. Stable isotope analysis is a common technique for analyzing foraging ecology and we outline our rationale for why it is appropriate for quantifying sex differences in feeding below. Our investigation uses a meta‐analytic approach, synthesizing previously published stable isotope data on vertebrates, with a global geographical scope.

### 1.4 Stable isotope ecology

Over recent decades, stable isotope analysis has become an effective tool used for investigating animal ecology (Hobson, 1999; Hobson & Welch, 1992; Swan et al., 2020). Because the ratios of naturally occurring stable isotopes vary in the foods animals consume, and these ratios are incorporated into animal tissues during formation, much can be revealed about an animal's ecology by analyzing stable isotope ratios in its different tissues (Ponsard & Arditi, 2000). Different isotopic systems provide alternative information about the animal from which they are sampled (Newton, 2016), such that ratios of nitrogen‐stable isotopes (δ ^15^N, see Methods for an explanation of δ notation) vary with trophic levels (Caut et al., 2009) and ratios of carbon‐stable isotopes (δ ^13^C) vary with food chain basal resource (Farquhar et al., 1989; Yoneyama et al., 2010). For example, relative ^15^N enrichment of polar bears compared with seals indicate polar bears occupy a higher trophic level (Hobson et al., 2002) and δ^13^C can distinguish the diets of zebras and giraffes that feed on C4 and C3 plants, respectively (Codron et al., 2006). Combined stable isotope ratios of animal tissues thus allow inferences about the individual niche, meaning ecological differences can be quantified at various levels, including niche differences between males and females, with the greater sex differences in stable isotope ratios taken to indicate more ecological divergence (Foote et al., 2012; Lehmann et al., 2015).

### 1.5 Is size dimorphism associated with isotopic sex differences?

Because stable isotope data tend to be reported reasonably consistently across taxa, compared with other measures of diet, the considerable stable isotope ecology literature provides an opportunity to investigate cross‐species associations between sexual dimorphism and ecological (isotopic) sex differences. Our study achieves three main aims. First, using meta‐analytic models, we quantify between‐study variation in isotopic sex differences in the stable isotope literature and the fraction of this variation constituting heterogeneity (*I* ^2^). In the context of meta‐analysis, heterogeneity describes the amount of observed between‐study variation in effect size that is due to nonrandom variation in true effect size, as opposed to random sampling variation (Borenstein et al., 2017). As a consequence, heterogeneity also indicates the fraction of between‐study variation that may be explained by predictor variables, such as the traits of study subjects. Second, having discovered substantial heterogeneity among studies, we next investigate how much heterogeneity in isotopic sex differences can be explained by size dimorphism. We use meta‐regression models including size dimorphism as a predictor variable, to examine associations with isotopic sex differences, and interpret the strength of associations as an indicator of the amount of heterogeneity in ecological sex differences that are explained by sexual dimorphism.

We use size dimorphism as a predictor variable in our analyses despite our interest in its response to certain ecological contexts. Our choice is primarily pragmatic: the diversity of the stable isotope literature means we can readily compute effect sizes using means, errors, and sample sizes for isotopic data of both sexes in many species. In contrast, body size data are most commonly available as mean values, and therefore more suitable as a predictor variable. Our choices also moderate the sensitivity of our meta‐analyses. Stable isotopes may not capture all ecological differences between sexes; for example, male and female birds may feed on different seeds, which would not manifest as trophic level differences. Similarly, the sexes may differ in trophic structures and feed on different diets, while being the same body mass, which would be missed by our measure of size dimorphism. However, our sacrifice of some of this detail allowed us to maximize the taxonomic scope and therefore the generality of our results. It also means that our meta‐analyses are conservative in nature and that associations between sexual dimorphism and ecological sex differences may be stronger than we detect here.

### 1.6 How important is the ecological context?

Our final aim was to examine whether associations between sexual dimorphism and ecological sex differences are modified by the ecological context. We first test whether species’ dietary class and/or mean species size modify relationships between size dimorphism and isotopic sex differences. Dietary class may modify the impact of size dimorphism on isotopic sex differences because species consuming different diets vary in their ecological flexibility. For example, omnivores by definition feed at more trophic levels than herbivores, which could create more opportunity for size dimorphism to exert an influence on the trophic level of each sex. Mean species size (defined here as the mean of males and females) may influence the effect of size dimorphism by causing between‐species differences in resource access, which may then impact how size dimorphism affects resource use within species. For example, if size affects the maximum prey size available to each sex, size dimorphism may have a greater impact on smaller species that are already more limited concerning the size of their prey. Conversely, the greater absolute size of larger species may mean proportional size differences between males and females have more impact on their respective interactions with other food web members. We quantify the potential influence of species’ dietary class and mean size by including them as additional predictor variables, alongside size dimorphism, in meta‐regression models.

Another possibility is that size dimorphism has the greatest ecological impact on carnivores that are gape‐limited, meaning they can only consume prey smaller than themselves (Shine, 1991; Shine et al., 2003). For example, in an aquatic food chain formed of gape‐limited fish, each species can consume all species smaller than itself, but no species the same size or larger. A trophic level should therefore closely track body size, with the largest fish at the highest trophic level. If a fish species in such a food chain were size dimorphic, the larger sex would have greater access to larger, higher trophic level prey, than the smaller sex, resulting in a difference between males and females in the maximum possible trophic level. As optimal foraging theory predicts that predators often feed preferentially on larger prey, due to greater energetic returns per prey item (Dodrill et al., 2021; Stephens & Krebs, 1986), the larger sex in a dimorphic gape‐limited fish would be predicted to feed at a higher trophic level. Conversely, nongape‐limited predators and scavengers, such as cats, can consume prey orders of magnitude larger than themselves, which may minimize any impact of size dimorphism on the trophic levels of each sex. Therefore, we investigate whether gape limitation strengthens associations between size dimorphism and isotopic sex differences. We do this using a meta‐regression on a data set constrained to fish and snake species, which are presumed to be able to feed solely by swallowing whole prey and thus considered gape‐limited. The predictions from this model are then compared with those from a model containing all other carnivores in our data set, to assess whether the effect of size dimorphism on trophic sex differences is greater in gape‐limited carnivores.

## 2 METHODS

Our meta‐analytic approach and reporting were completed with reference to the guidelines laid out by O'Dea et al. (2021).

### 2.1 Data collection

We collated peer‐reviewed literature available in the Web of Science Core Collection. The stable isotope literature is large, with the search term “stable isotope” returning ~76,500 studies at the time of writing. To constrain the search, we combined the following specific terms, using the default publication year range of 1900–2020, on 10/11/2020: Isotop* Nich; Isotop Nich* Male; Isotop* Nich* Female; Isotop* Nich* Male Female; Isotop* Nich* Sex Diff*; Isotop Nich* Dimorph; Isotop Dimorph*.

**FIGURE 1:**
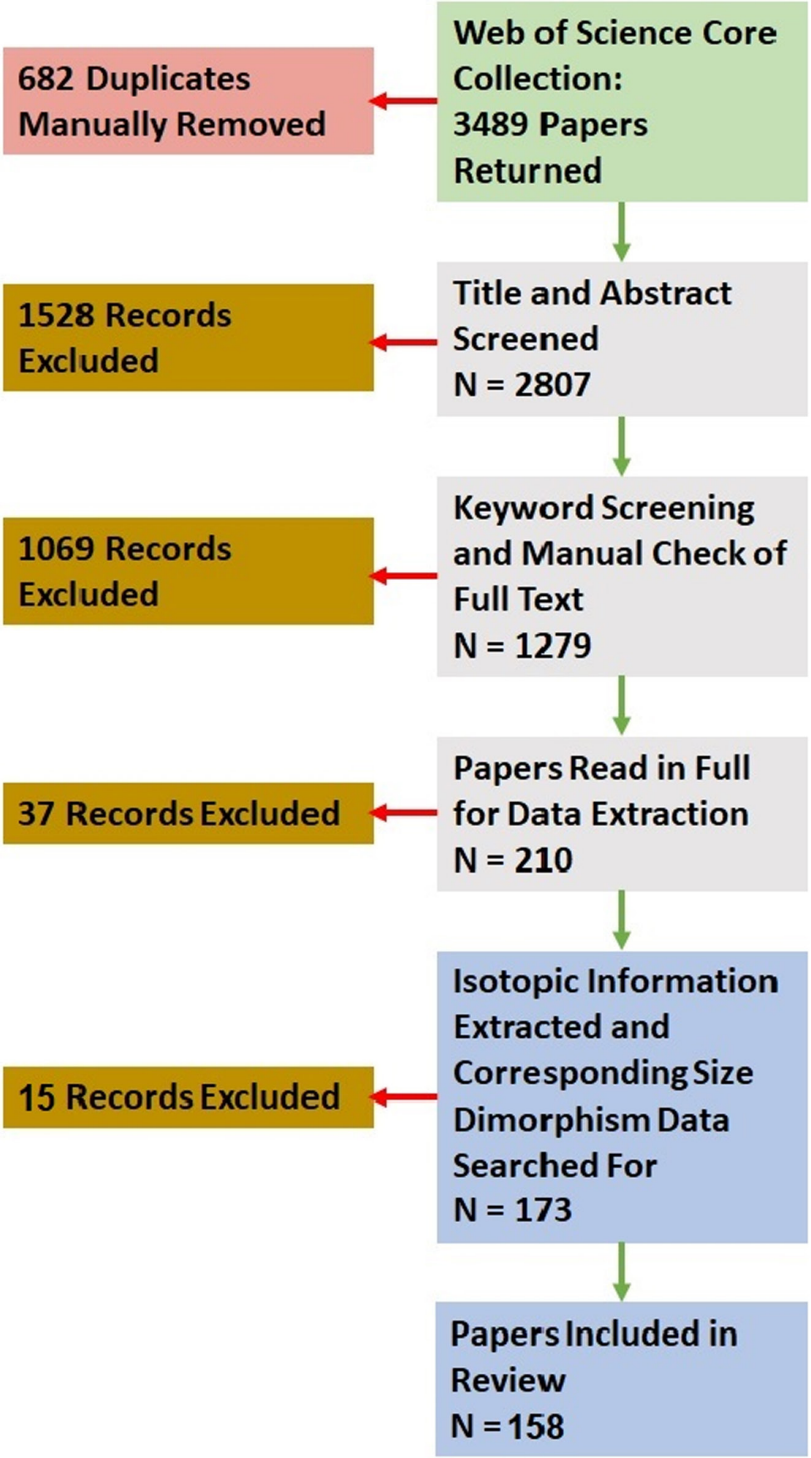
Our sequence of study collation, screening, and data extraction, alongside the number of studies excluded at each stage and included in the final analysis.

Our searches returned 3489 studies, which we placed into a spreadsheet to highlight duplicates for manual removal. Removing duplicates resulted in 2807 studies for the title and abstract screening. At this stage, we made the decision to constrain our analysis to the nitrogen and carbon stable isotope systems, due to the relatively small number of studies using other systems that were returned by our search terms. We also rejected studies during the title and abstract screening if they did not use bulk stable isotope analysis, used samples of human, museum, archeological or palaeontological origin, were review, comment, or method papers, or if the animals sampled were not wild, not adults, not vertebrates or if data were not available for both sexes. We then searched the remaining 1279 studies using the ctrl + F search function and, separately, the terms “sex”, “male” and “female”, excluding studies if they contained none of these terms, under the assumption that they did not contain stable isotope ratios for each sex and, if at least one term was present, checking for the presence of the required data. Additional reasons for exclusion were if the full text was inaccessible without purchase or contacting authors, presented incomplete data (mean, error, or sample size missing), was not in English, or was a paper correction. We then attempted to extract data from the remaining 210 studies. Additional reasons for exclusion at this stage were if raw data were presented as images with >50 rows, if data were from an earlier study already included or if data extraction from figures was not possible. We extracted data from figures using a mouse pointer to individually select data points from an image of the figure, with the image calibrated to the axis values from the original figure; therefore, too much point overlap made this process inaccurate, because not all points could be selected for inclusion. The entire process provided 173 studies in which mean, standard deviation, and sample sizes for each sex were presented in the manuscript, or could be calculated from raw data, or could be taken from model outputs, or extracted from figures (Figure 1). We collected data for any vertebrate species, from any global location and, if stable isotope ratios for each sex were presented for more than one tissue type, we entered each tissue as a separate row in our database.

### 2.2 Effect size choice and calculation

All the stable isotope data we collected were presented in permil units (‰). Permil units describe enrichment or depletion of heavy isotopes, relative to international standards, which exist for nitrogen, carbon, and all other commonly used systems in stable isotope analysis. For example, the standard for nitrogen‐stable isotope ratios is atmospheric nitrogen (air). The relative enrichment or depletion is expressed using delta (δ) notation, such that

$$ẟ=\frac{\left(R_{\text{sample}-}R_{\text{standard}}\right)}{R_{\text{standard}}}$$

where *R*_sample_ equals the isotope ratio of the sample and *R*_standard_ equals the isotope ratio of the standard (McKinney et al., 1950) where *R* = heavy isotope/light isotope, for example, ^15^N/^14^N. Thus, a positive *ẟ* value indicates enrichment in the heavier isotope (in this case ^15^N) and a negative value indicates isotopic depletion of the sample, relative to the international standard. When comparing two sampling units, such as sex, a more positive (or less negative) value for one sex indicates enrichment in the heavy isotope relative to both the standard and the other sex. It is this difference between sexes in isotopic enrichment that we have used to calculate the effect sizes in our meta‐analysis.

All studies from which we extracted data expressed stable isotope data in permil units, therefore further standardization of effects sizes was not necessary (Nakagawa & Cuthill, 2007; Nakagawa & Santos, 2012). We calculated mean differences between male and female stable isotope ratios as the raw mean difference between isotopic means of each sex, as found in each study. We calculated these with a positive mean difference indicating that males were ^15^N or ^13^C enriched compared with females and a negative mean difference indicating females were isotopically enriched compared with males. For example, a positive ẟ^15^N mean difference indicates that males feed, on average, at a higher trophic level.

Regarding meta‐analyses of variation, two effect size measures are often recommended in the fields of ecology and evolution, which are the log Variability Ratio (lnVR) and the log Coefficient of Variation Ratio (lnCVR; Senior et al., 2020). lnCVR has the advantage of allowing for mean–variance relationships in effect sizes (i.e., an increase in variance with mean value; Senior et al. 2016): our data did not show any evidence of such relationships (Appendix 1). In addition, because lnCVR accounts for variation in mean value by expressing absolute variation as a proportion of group mean, sex differences in isotopic variation could actually be misrepresented through this standardization. For example, if one sex is twice as enriched relative to the international standard as the other and shows twice as much variation, lnCVR would express this as an equal variation. Conversely, as lnVR is calculated using the raw variation values for each group, with no accounting for mean values, the more enriched sex would also be shown to be twice as a variable, more accurately representing each sex's ecology. We, therefore, selected lnVR as our effect size metric for sex differences in variation.

Finally, we selected mass dimorphism (kg) as our measure of size dimorphism, as this measure was most readily available for the highest number of vertebrates, allowing the taxonomic scope of our analysis to be as wide as possible. Body masses for each sex were established using web searches, prioritizing data from peer‐reviewed scientific studies, followed by published books and, if necessary, taxon‐specific websites. As reliable body mass data could not be obtained for all species, the number of studies in the analysis was reduced to 158. A list of data sources used in our meta‐analyses is provided in the Data Sources section.

### 2.3 Model choice and structure

All models used complete case analysis, meaning rows containing missing data for predictor variables or effect sizes (dependent variables) were removed from the analysis. We used multilevel meta‐analytic models to quantify the amount of between‐study variation that exists for each isotope and effect size and how much of this variation constitutes heterogeneity, as opposed to random sampling variation. We then used multilevel meta‐regression models to investigate whether the heterogeneity found could be explained by our predictor variables. To investigate the relationship between sexual dimorphism and ecology, we examined the strength of associations between size dimorphism and sex differences in isotopic mean and variance for carbon and nitrogen.

To examine whether ecological context can modify the relationship between dimorphism and ecology, we ran meta‐regressions using sex differences in mean nitrogen isotope as the response variable, with additional predictor variables, dietary class, and mean species size, included alongside size dimorphism. Dietary class constituted a categorical variable with three levels: carnivore, omnivore, and herbivore. Mean size was a continuous variable, calculated by averaging the male and female mass data used for calculating size dimorphism. We ran models including dietary class and mean size separately and together, with and without all combinations of two‐ and three‐way interactions. Three‐way interactions were theoretically justified because, if mean species size could modify the effect of size dimorphism on the trophic level, this modification may be more apparent in species with more inherent trophic flexibility, such as omnivores, than those with less, such as herbivores. In addition, the sample size was large enough that the number of parameters to be estimated with three‐way interactions did not prevent models from converging. The best models were identified using AIC_c_ scores, with lower scores taken to indicate better models (Arnold, 2010).

Initially, we only considered a modifying effect of dietary class on nitrogen mean sex differences because carbon sex differences did not exhibit a significant association with size dimorphism across our entire data set. However, this could be expected to exclude herbivores, which likely feed at only one trophic level, from showing an association. We, therefore, conducted an additional model examining whether dietary class modified the association between size dimorphism and sex differences in mean carbon stable isotope ratio. This model balances our analyses because carbon isotopes in terrestrial systems are primarily influenced by plant photosynthetic mechanisms and therefore represent a niche axis along which the sexes could more easily diverge in herbivores.

As a final test of the association between size dimorphism and ecological sex differences, we quantified the effect of gape limitation by running two meta‐regressions including only size dimorphism as a predictor variable and limiting the data sets to gape‐limited and nongape‐limited carnivores, in which size dimorphism may have differing impacts on sex differences in trophic level.

Residuals of all models were approximately normally distributed; thus, no data transformations were used.

### 2.4 Random effects

In all the above multilevel models, we included study identity and species as random factors, to account for random sampling variation at both these levels and to adequately account for pseudoreplication, since we potentially considered measures for several tissue samples from the same specimens. We also included phylogeny as a random factor, to account for relatedness between the species included in our data set, following the method of Sanchez‐Tojar et al. (2020).

### 2.5 Publication bias and sensitivity analysis

Scientific literature may be subject to publication bias, whereby favorable results are preferentially published, thus skewing the results of meta‐analyses. We produced funnel plots to identify such biases, by visualizing the distribution of published effect sizes and determining whether there are missing observations that might be expected in the literature (based on variation in effect sizes). In addition, biases may arise when research builds upon influential results from poor quality or low power studies, leading to reduced effect sizes through time, as the true effect is quantified with repetition or higher quality studies. To test for such publication bias in isotopic sex differences, we ran meta‐regressions using sex differences in isotopic mean and variance, for carbon and nitrogen, as the dependent variable and publication year as the only predictor variable.

The results of meta‐analyses may also be sensitive to decisions about the weights assigned to individual studies as well as to high‐influence data points (Koricheva et al., 2013). Meta‐analytic models usually account for both within‐study variance and between‐study variance when assigning weights to individual study results. However, when between‐study variance is high, within‐study variance can be masked when weighting studies, potentially impacting model results. We, therefore, ran additional models using only the inverse of within‐study variance to assign study weights, to determine the influence of our choice of weighting parameter. Finally, to analyze the sensitivity of our models to high‐influence data points, we completed a leave‐one‐out analysis, to calculate Cook's distances for each data point and ran additional models with high‐influence data points removed. The results of our tests of publication bias, alternate study weighting and sensitivity analysis, alongside justifications for final data inclusion and model choices can be found in Appendix 1.

### 2.6 Software

All data processing, analyses, and plotting were completed using R v4.0.2. We used the R package “metaDigitise” v1.0.1 (Pick, Nakagawa & Noble, 2019) for all data extraction from figures and the package “metafor” v.2.4‐0 (Viechtbauer, 2010) to calculate all effect sizes and to run all meta‐analytic and meta‐regression models. Our phylogeny was constructed using the “rotl” v3.0.12 (Michonneau et al., 2016) and “ape” v5.6.2 (Paradis & Schliep, 2019) packages and we calculated the phylogenetic signal with “phylosignal” v1.3 (Keck et al., 2016) and “phylobase” v0.8.1 (Hackathon, 2020). We created all plots using the R package “ggplot2” v3.3.3 (Wickham, 2016) and tables using “flextable” v0.7.3 (Gohel et al., 2020).

## 3 RESULTS

### 3.1 Data set

Our final database contained isotopic information from 158 studies, covering 163 species. Mammals were the most common taxa (*n* = 68), followed by birds (*n* = 60), fish (*n* = 18), reptiles (*n* = 17), and a single amphibian. The species with the greatest female‐biased dimorphism was the northern map turtle (*Graptemys geographica*), in which females are 10× the mass of males and the species with the greatest male‐biased dimorphism was the elephant seal (*Mirounga leonina*), with males seven times larger than females. The number of effect sizes used in the analyses was highest for δ ^15^N mean sex differences (*n* = 282), followed by δ ^13^C mean differences (*n* = 276), δ ^15^N lnVR (*n* = 272), and δ ^13^C lnVR (*n* = 266).

### 3.2 Quantifying heterogeneity in between‐sex isotopic differences

**FIGURE 2:**
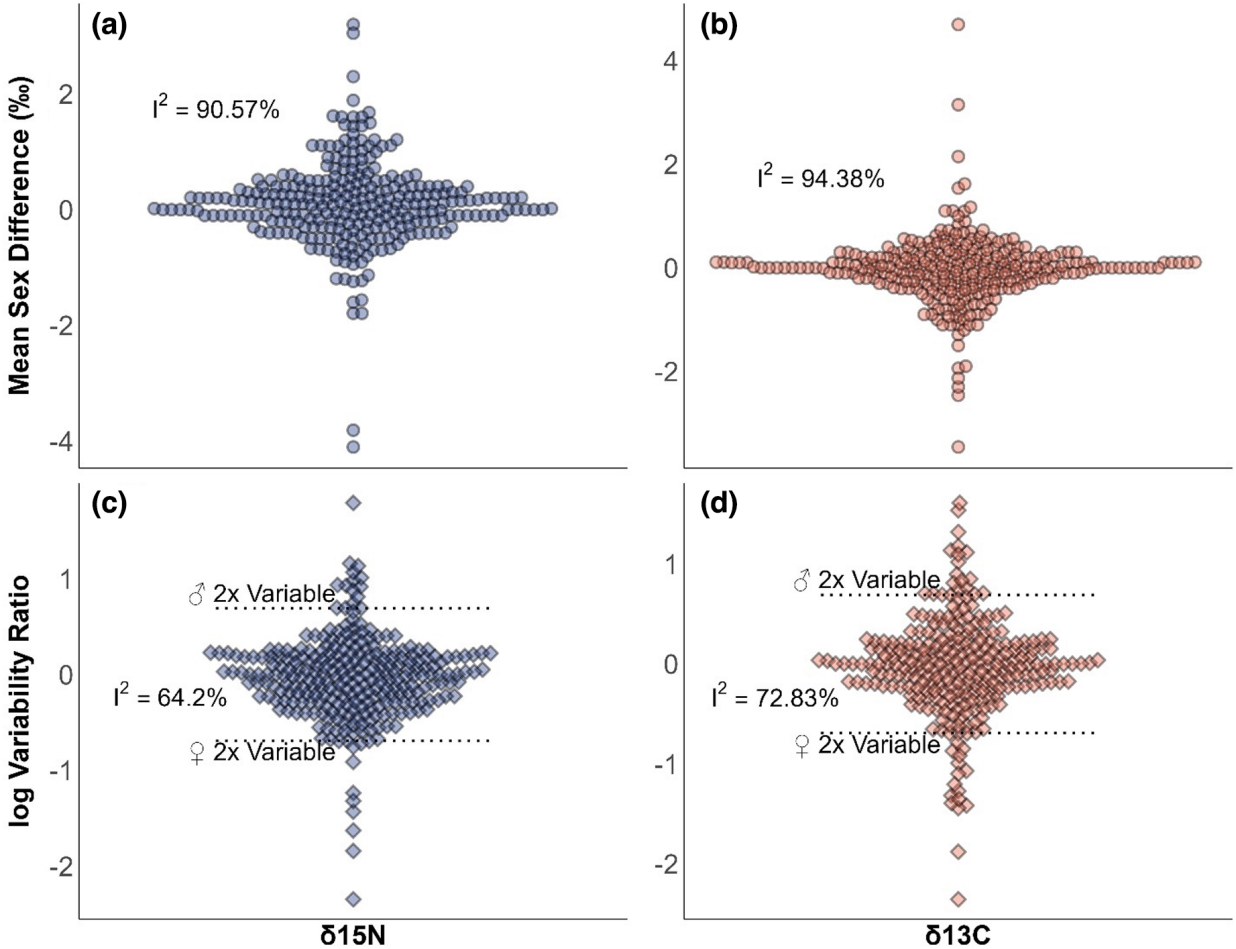
Published stable isotope ratio sex differences for nitrogen mean (a), carbon mean (b), nitrogen variation (c), and carbon variation (d). In (a, b), each point represents the raw difference between male and female mean stable isotope ratio, of one tissue of one species. Positive values indicate higher values in males, whereas negative values indicate higher values in females. In (c, d), each point represents the log male:female variability ratio of one tissue, in one species. Positive values indicate males showed more isotopic variation and those above the dotted line indicate that males were more than twice as variable as females. Negative values indicate females showed more isotopic variation and those below the dotted line indicate that females were more than twice as variable as males. *X*‐axes constitute one category, with jitter added to better visualize overlapping observations.

Between‐study variation was found for sex differences in mean δ ^15^N (trophic level) and δ ^13^C (food chain basal carbon resource) and sex differences in δ ^15^N and δ ^13^C variation (Figure 2). The amount of heterogeneity (*I* ^2^) was 90.57% and 94.38% for δ ^15^N and δ ^13^C mean sex differences, respectively. Such high heterogeneity indicates that almost all between‐study variation in effect size is nonrandom and has the potential to be explained by predictor variables. Regarding sex differences in isotopic variation, heterogeneity was 64.2% and 72.83% for δ ^15^N and δ ^13^C, respectively, indicating that the majority of between‐study variation in between‐sex differences in an isotopic variation also has the potential to be explained by predictor variables. In the case of nitrogen, one sex was at least twice as variable as the other in 8.5% of cases and for carbon, in 13.1% of cases (Figure 2).

### 3.3 Associations between isotopic sex difference and size dimorphism

**FIGURE 3:**
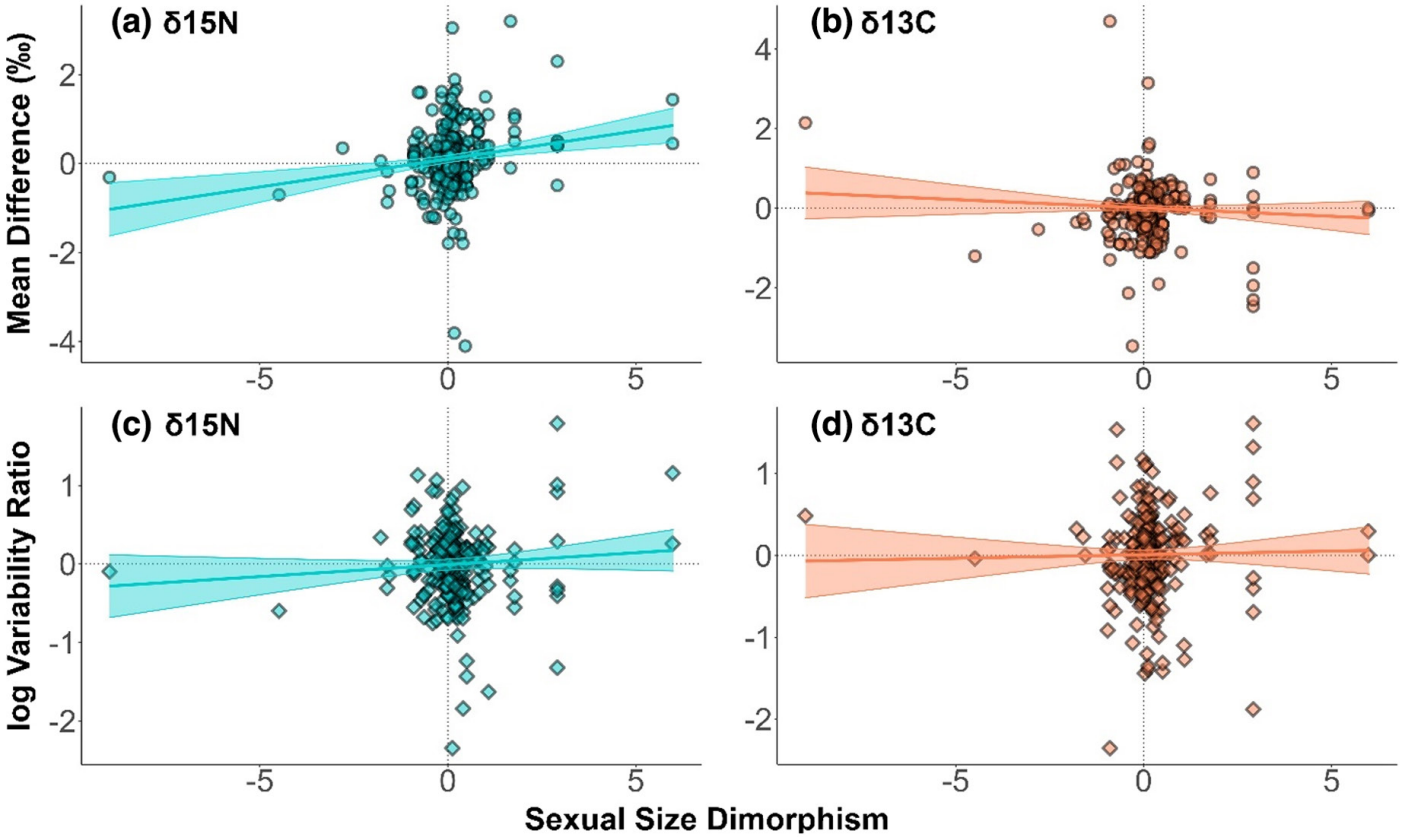
The relationship between sexual size dimorphism and stable isotope sex differences in nitrogen mean (a), carbon mean (b), nitrogen variation (c), and carbon variation (d). Lines and 95% confidence intervals are based on meta‐regression predictions. Data points are raw data, overlaid to visually assess how well size dimorphism explains isotopic sex differences.

To examine the possibility that size dimorphism relates to ecology, we carried out meta‐regressions containing size dimorphism as the sole predictor variable and found modest or nonexistent associations with isotopic sex differences. The estimated effect size of size dimorphism on δ ^15^N mean difference was significantly positive (mean = 0.126, 95% CI: 0.06–0.19, *p* = <.001), indicating that a size dimorphism of 100% led to a δ ^15^N increase of 0.126‰, on average. This effect was modest compared with the variation in isotopic sex differences in our data set (range δ^15^N sex difference: −4.1 to 3.2‰). Models of relationships between size dimorphism and δ ^13^C mean differences, δ ^15^N variation, and δ ^13^C variation produced estimate confidence bands that overlapped zero, indicating no significant associations between size dimorphism and these measures of isotopic sex differences. The predictions from these models, alongside their underlying raw data, are visualized in Figure 3.

### 3.4 Quantifying the effects of ecological context

**FIGURE 4:**
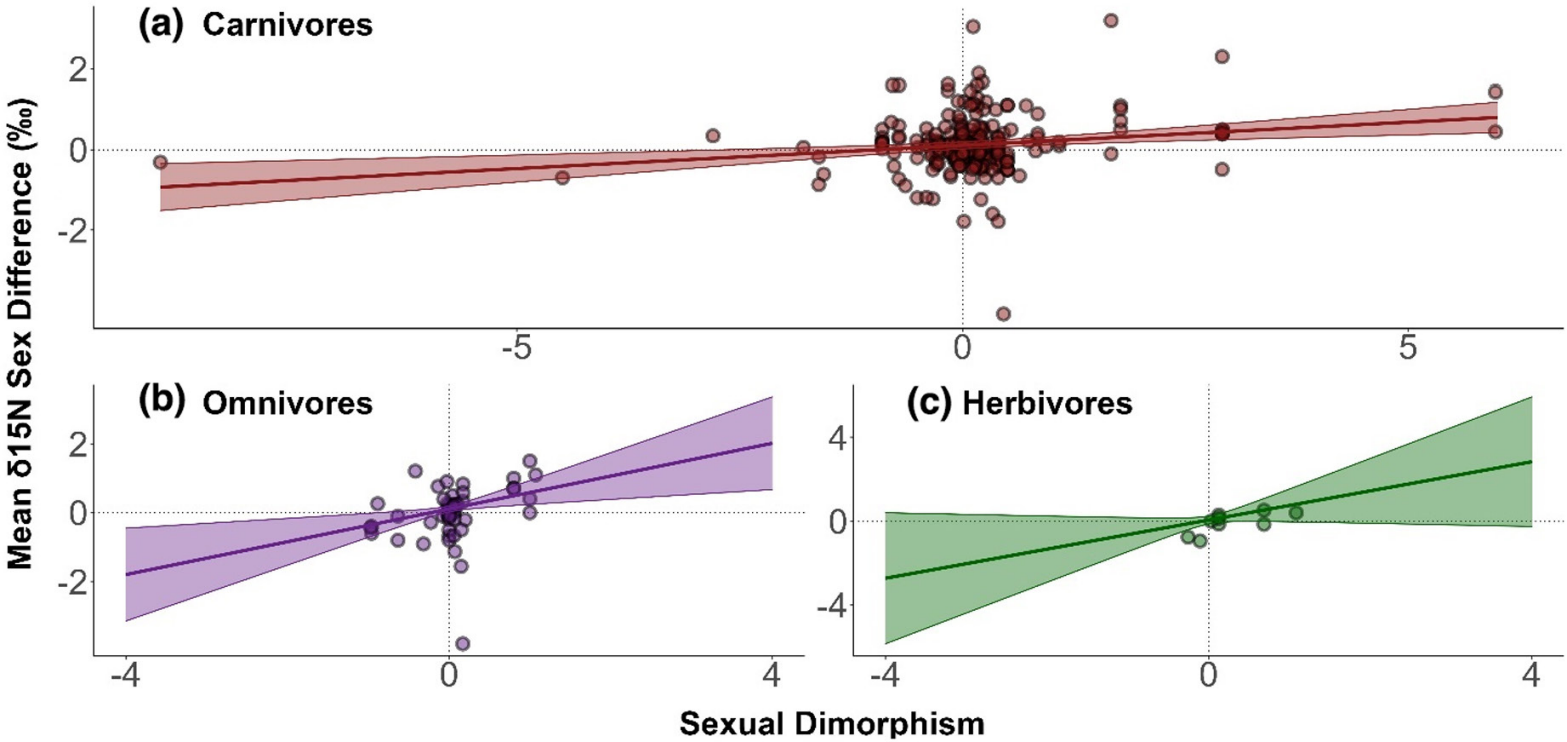
The relationship between sexual size dimorphism and mean nitrogen stable isotope ratio sex differences in carnivores (a), omnivores (b), and herbivores (c). Lines and 95% confidence intervals are based on meta‐regression predictions. Data points are raw data, overlaid to visually assess how well size dimorphism explains trophic sex differences in each dietary class.

To test the possibility that associations between size dimorphism and feeding vary in strength among ecological contexts, we used meta‐regressions to test whether dietary class, mean size, or gape limitation modified the effect of size dimorphism on isotopic sex differences. Model selection using AIC_c_ scores indicated that a model containing species mean size and dietary class as predictors of δ^15^N sex differences, with an interaction between size dimorphism and dietary class, improved model fit (AIC_c_ = 512), compared with the size dimorphism‐only model above (AIC_c_ = 518; Table 1). The best model contained an interaction between size dimorphism and dietary class, such that the association between size dimorphism and sex differences in nitrogen mean was statistically nonsignificant in herbivores, significant and moderate in carnivores (0.17, 95% CI: 0.053–0.18, *p* < .001), and significant and strongest in omnivores (0.36, 95% CI: 0.019–0.7, *p* = .038; Figure 4). The model also contained a significant effect of mean species size on sex differences in nitrogen mean (0.0000036, CI: 0.00000023–0.0000069, *p* = .036).

**TABLE 1:** AIC scores for models examining associations between size dimorphism and δ ^15^N mean sex differences in different ecological contexts.

Model formula	AIC
δ ^15^N Mean Sex Difference ~ Size Dimorphism*Dietary Class + Species Mean Size	512.06
δ ^15^N Mean Sex Difference ~ Size Dimorphism*Species Mean Size + Dietary Class	513.28
δ ^15^N Mean Sex Difference ~ Size Dimorphism*Species Mean Size	513.89
δ ^15^N Mean Sex Difference ~ Size Dimorphism*Dietary Class*Species Mean Size	513.89
δ ^15^N Mean Sex Difference ~ Size Dimorphism*Dietary Class	514.36
δ ^15^N Mean Sex Difference ~ Size Dimorphism + Dietary Class*Species Mean Size	515.16
δ ^15^N Mean Sex Difference ~ Size Dimorphism + Dietary Class + Species Mean Size	515.32
δ ^15^N Mean Sex Difference ~ Size Dimorphism + Species Mean Size	515.91
δ ^15^N Mean Sex Difference ~ Size Dimorphism + Dietary Class	517.64

**FIGURE 5:**
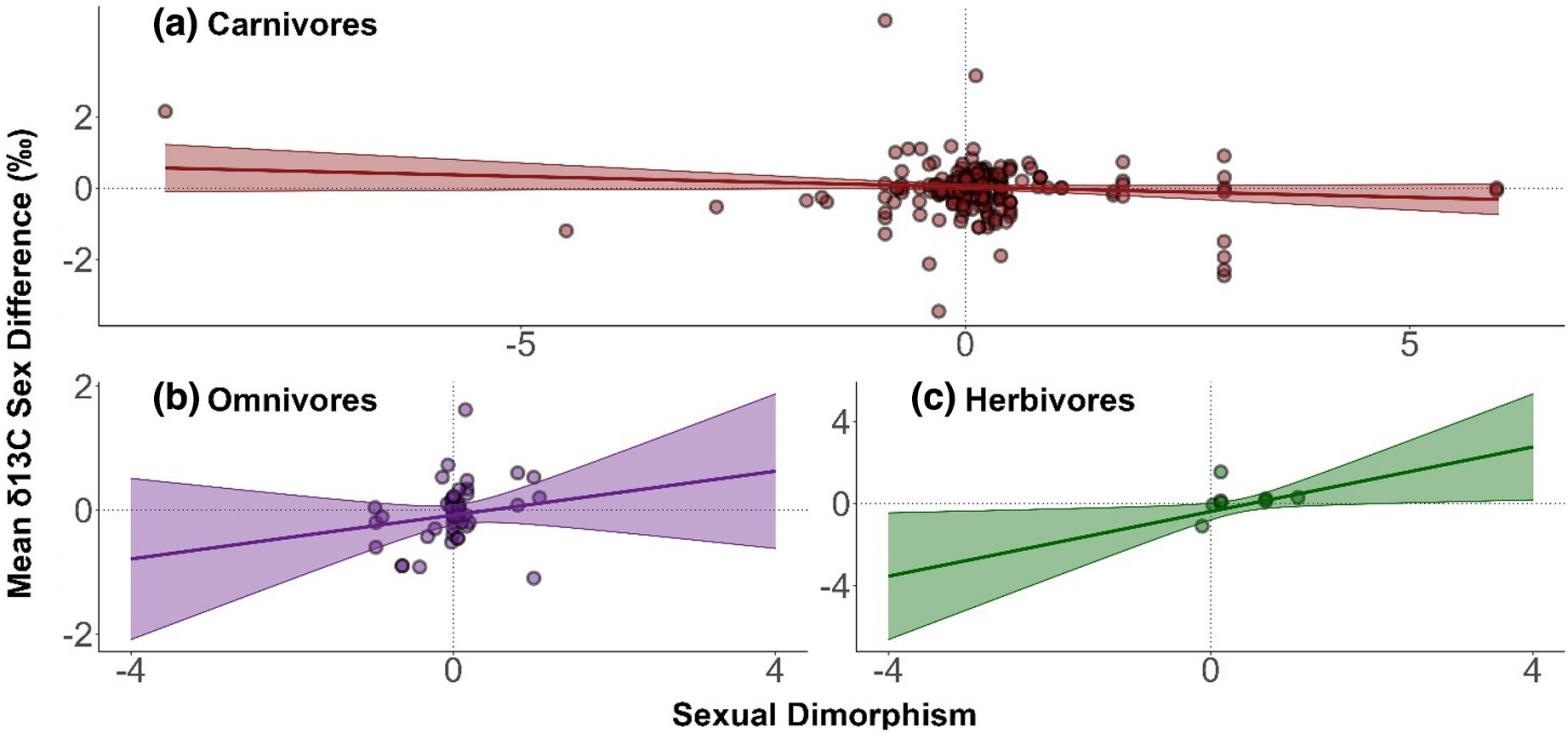
The relationship between sexual size dimorphism and mean carbon stable isotope ratio sex differences in carnivores (a), omnivores (b), and herbivores (c). Lines and 95% confidence intervals are based on meta‐regression predictions. Data points are raw data, overlaid to visually assess how well size dimorphism explains trophic sex differences in each dietary class.

Regarding sex differences in δ ^13^C, including diet alongside size dimorphism produced results that contrasted with δ ^15^N. In the case of carbon, carnivores and omnivores instead exhibited nonsignificant associations between size dimorphism and isotopic sex differences, whereas herbivores exhibited a significant positive association (0.847, 95% CI: 0.139–1.555, *p* = .02; Figure 5).

**FIGURE 6:**
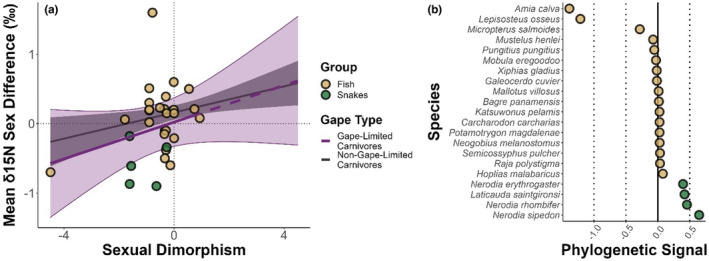
(a) The relationship between sexual size dimorphism and mean nitrogen stable isotope ratio sex difference in gape‐limited and nongape‐limited carnivores. Lines and 95% confidence intervals are based on meta‐regression predictions. Data points are raw data for gape‐limited carnivores, overlaid to assess how well size dimorphism explains trophic sex differences in gape‐limited carnivores. Predictions based on gape‐limited species are shown in purple (the prediction line is dashed outside the raw data range) and for the nongape‐limited carnivores in gray. (b) Local indicators of phylogenetic association (phylogenetic signal) for mean nitrogen sex differences in gape‐limited carnivores.

Limiting the data set to only gape‐limited predators, in which trophic level is predicted to relate more closely to body size, resulting in a 41% increase in the estimated effect of size dimorphism on δ ^15^N mean difference. However, the effect was nonsignificant because of the more modest sample size (0.133, 95% CI: −0.0412 to 0.306, *p* = .135), relative to nongape‐limited (0.094, 95% CI: 0.023–0.17, *p* = .01; Figure 6a). Our data set contained local phylogenetic signals for δ ^15^N sex differences in gape‐limited predators, with positive phylogenetic signal in snake species (Figure 6b), controlling for which weakened the association between size dimorphism and trophic sex differences in gape‐limited predators. A nonphylogenetic meta‐regression showing a stronger association may be found in Appendix 1.

## 4 DISCUSSION

Using meta‐analytical methods, we examined cross‐species relationships between sexual size dimorphism and ecological divergence between sexes, measured using stable isotopes. We found that across 166 globally distributed species, size dimorphism was weakly associated with sex differences in mean trophic level (δ^15^N), but not mean food chain basal resource (δ^13^C), or variation in either isotope. We also found associations between size dimorphism and isotopic sex differences to be modified by ecological context. The effect of size dimorphism on sex differences in mean trophic level was strongest in omnivores, more modest in carnivores, absent in herbivores, and very modestly affected by species mean size. Dietary class influenced the effect of size dimorphism on sex differences in food chain basal resource in an inverse manner to trophic level, as an association was found in herbivores, but not in omnivores or carnivores. Finally, we found partial evidence that sex differences in trophic level could be more strongly associated with size dimorphism in gape‐limited than in nongape‐limited carnivores, as predicted by theory.

### 4.1 Heterogeneity exists in isotopic sex differences

We found heterogeneity in between‐sex differences in isotopic mean and variance, for nitrogen and carbon isotopes. Heterogeneity in sex differences for δ ^15^N and δ ^13^C indicates nonrandom between‐study variation in the extent to which males and females feed at different trophic levels and in different food chains, respectively. Our meta‐analytic models, therefore, indicated that large amounts of between‐study variation in ecological sex differences may be explained by study‐level variables. Though we found some isotopic sex differences to be associated with size dimorphism, which we discuss presently, a large amount of variation was unexplained. Though we found some isotopic sex differences to be associated with size dimorphism, which we discuss presently, a large amount of variation was unexplained. This unexplained variation remains open to explanation by further analyses, some suggestions for which we also outline below. We hope our database provides a useful starting point for further investigations of sexual dimorphism and ecological differences between males and females (Dryad: https://doi.org/10.5061/dryad.k98sf7m99).

### 4.2 Across all species, size dimorphism is associated with sex differences in trophic level, but not food chain basal carbon resources or ecological variability

We found evidence that sexual size dimorphism does, in some instances, scale positively with ecological sex differences. Size dimorphism exhibited a moderate positive association with sex differences in mean δ ^15^N and thus explained some variation in trophic level differences between males and females. Previous cross‐species investigations of the relationship between size dimorphism and δ ^15^N sex differences have often found inconsistencies, with size dimorphism sometimes relating to trophic differences and sometimes not (Mancini et al., 2013; Phillips et al., 2011). These inconsistencies are likely because the effect is probably modest and may be modified by a wide range of factors, as evidenced by the amount of unexplained variation in our data set. Thus, the size and scope of our analysis is likely the factor that has allowed us to find a clear but moderate effect of size dimorphism on trophic‐level differences between sexes. No relationships existed between size dimorphism and variation in δ ^13^C mean sex differences across all species or sex differences in variation in either isotope. Size dimorphism thus does not appear to have a general cross‐species influence on sex differences in food chain basal carbon resources or trophic variability. These results conflict somewhat with the conclusions of studies on individual species (Calado et al., 2020; Voigt et al., 2018). It may therefore be the case that size dimorphism is related to sex differences in basal carbon resources and trophic variability in particular species, populations, or contexts, but this association is not sufficiently consistent to covary with sex differences across species.

### 4.3 Associations between size dimorphism and isotopic sex differences are modified by dietary class and gape limitation, but not mean size

We found that ecological context influenced associations between size dimorphism and ecological sex differences. Dietary class modified the effect of size dimorphism on trophic‐level (δ^15^N) differences between males and females, with no effect of dimorphism in herbivores, a moderate effect in carnivores, and the strongest effect in omnivores. In contrast, we found an association between size dimorphism and sex differences in food chain basal resource (δ^13^C) in herbivores, but not in omnivores or carnivores. These results are consistent with our prediction that inherent differences in ecological flexibility might alter the association between size dimorphism and ecology and make sense given the respective feeding niches of the three dietary classes.

As herbivores would be expected to feed exclusively on plants, they should only occupy the position of the primary consumer, leaving little scope for size dimorphism to influence trophic level. Instead, herbivores can more easily diverge along the niche axis related to plant consumption. Because most herbivores in our data set occupy terrestrial habitats, plant consumption is reflected in carbon isotopes in their tissues. Thus, if size dimorphism relates to dietary divergence in herbivores, this divergence seems to arise as sex differences in plant consumption, as opposed to trophic level. For example, stable isotopes suggest that male African elephants (*Loxodonta africana*) consume more grass than females, which may be because larger body size increases food digestive efficiency and food ingestion per mouthful (Shannon et al., 2013).

Carnivores may occupy any position from secondary consumer upwards, meaning each sex could take prey from one or multiple trophic levels, creating some opportunity in carnivores for trophic differences between the sexes to be influenced by size dimorphism. In contrast to the other two groups, omnivores would be expected to consume foods from a minimum of two trophic levels, leading to a greater probability of trophic differences between sexes and the largest opportunity for size dimorphism to influence this difference. This might be why size dimorphism was most strongly associated with trophic sex differences in omnivores in our data set. Sex differences in carnivore and omnivore basal resources may have covaried less with size dimorphism because the basal resources of nonprimary consumers relate not only to their diet but also to the diets of their prey (Codron et al., 2018). Therefore, unless dimorphism produces sex differences in prey access that corresponds to different basal resources, for example, one sex exclusively hunting browsers and the other hunting grazers, a strong association between dimorphism and basal resource sex differences is unlikely. The indirect nature of the relationship between size dimorphism and basal resource in carnivores and omnivores could mean that divergence along this niche axis is less likely to be driven by dimorphism than in herbivores. Together, these results suggest that the association between size dimorphism and ecological sex differences is dependent on the ecological opportunity for feeding differences between males and females.

Our model also contained an independent effect of species mean size on sex differences in trophic levels. However, this effect was multiple orders of magnitude lower than the analytical reproducibility of nitrogen stable isotope analysis. Thus, even though the model estimate was technically positive, we conclude that absolute species size has no meaningful effect on trophic‐level sex differences.

Limiting the data set to gape‐limited carnivores led to a 41% increase in the effect of size dimorphism on trophic sex differences, compared with nongape‐limited carnivores, though the gape‐limited model was nonsignificant. There is substantial evidence available that larger gape increases maximum ingestible prey size and trophic level in fish and snakes (Barnes et al., 2021; Nilsson & Brönmark, 2000; Persson et al., 1996; Webb & Shine, 1993). Consequently, the larger effect of size dimorphism on trophic sex differences in the gape‐limited predators we analyzed could indicate that gape limitation increases the ecological relevance of dimorphism, producing a stronger relationship between dimorphism and ecology. More research is required, however, due to the nonsignificance of our model. The wide confidence intervals in the gape‐limited model may have been due to the small sample size (*n* = 30) and any potential interaction between gape limitation, size dimorphism, and ecological sex differences should therefore be examined with a larger data set.

An additional possibility is that gape limitation is important to the relationship between size dimorphism and ecological sex differences only when certain conditions are met. There was a phylogenetic signal for trophic‐level sex differences in our gape‐limited data (Figure 6b) and in nonphylogenetic models, the effect of size dimorphism in gape‐limited predators was significant and substantially greater than when controlling for phylogeny (Appendix 1). It could be the case that the phylogenetic random effect captured differences between species in the niches or food webs to which they have adapted. If gape limitation is to produce trophic sex differences in a species, then body size must relate to the trophic level in the prey of that species, in order for higher trophic level prey to be accessible to only the larger sex. This condition may not hold true in all taxa or food webs, and this wider context may need to be considered when investigating the influence of gape limitation on associations between sexual dimorphism and trophic sex differences.

In summary, we found the extent of size dimorphism to be associated with the extent of ecological sex differences, an association that increased in strength when the ecological opportunity for trophic variation was greatest and potentially when size dimorphism was more ecologically relevant. Our results are consistent with both a potential role for ecological character displacement in enhancing sexual dimorphism or for ecological sex differences to arise due to reproductive differences. However, as the associations we found were generally modest, they support previous predictions that the role of ecological character displacement is relatively minor (De Lisle & Rowe, 2015; Fairbairn, 1997) and suggest that size dimorphism produced via reproductive differences is not a powerful driver of feeding differences between males and females.

## 5 FUTURE RESEARCH DIRECTIONS

Several questions arise from our meta‐analysis that should form the subject of future work. First, the results of our analysis on gape limitation were uncertain and the apparent influence of phylogeny on our results could suggest that the relevance of gape limitation is species or niche specific. Future analyses could therefore examine the impact of gape limitation on ecological sex differences with a greater sample size and taxonomic scope than used presently. For example, a recent analysis found a positive relationship between gape size and fruit size consumed in frugivorous birds (McFadden et al., 2022), and including similar data in future analyses could allow the importance of gape limitation to ecological sex differences to be generalized across a wide range of species and niches.

As our results suggest a relationship between sexual dimorphism and ecology but do not distinguish between reproductive differences and ecological character displacement as driving that relationship, the next major question concerns their relative importance as evolutionary mechanisms. A weak role for ecologically mediated selection implies that sexual and fecundity selection are the main drivers of sexual dimorphism. However, a recent analysis found that size dimorphism was only weakly associated with sexual selection across species, leading the authors to suggest that “alternative mechanisms such as ecological character displacement may be crucial to understand the full diversity of [size dimorphism] in animals” (Janicke & Fromonteil, 2021). Considering our own results, alongside their conclusion, we suggest that a future priority should be the incorporation of sexual, fecundity, and ecologically mediated selection into single cross‐species analyses, to quantify their relative importance to the evolution of size dimorphism.

Our analyses support an association but suggest the cross‐species patterns may be modest. Why would ecological character displacement be a weaker selective force than other drivers of sexual dimorphism? One possibility is that the frequency‐dependent nature of resource competition means that the strength of competition falls as the sexes phenotypically diverge (De Lisle & Rowe, 2015). Alternatively, divergence from the species mean phenotype, while alleviating resource competition, may itself entail fitness costs that eventually exceed those of competition for resources (Bolnick & Doebeli, 2003; Slatkin, 1984). Either possible scenario may place an upper limit on the extent to which ecologically mediated selection can drive character displacement between sexes. Therefore, establishing the mechanistic limitations on ecological character displacement between sexes should also become the focus of future investigations, most likely via modeling and experiment.

A final question is what additional variables could be included in future analyses, to explain the considerable variation in ecological sex differences? Our analyses have highlighted that high amounts of between‐study variation in our data remain unexplained, providing opportunities to use our database to investigate additional drivers of ecological differences between males and females. Importantly, isotopic values for an animal's tissues may be affected by many factors, such as body size, body condition, diet quality, and ontogenetic growth (Carleton & Martinez del Rio, 2010; Lecomte et al., 2011; Wolf et al., 2009). Sex differences in any of these variables could potentially influence sex differences in isotopic signals and influence cross‐species isotopic comparisons. However, their impact is often species‐specific, so a comparative synthesis of the sort we have conducted would require species‐level data to become widely available across many taxa.

In relation to why ecological sex differences evolve, nutritional requirements are one potential avenue of investigation. Males and females may target distinct sets of resources in order to meet sex‐specific nutritional needs, such as lactation or sexually selected signals (Harrison et al., 2017; Thompson, 2013). These differences may influence foraging and other aspects of behavioral ecology (Morehouse et al., 2020). As stable isotopes in animal tissues vary with the foods animals consume, the sex differences in isotope ratio we have observed may illustrate how males and females target distinct resources, to fulfill their own sex‐specific nutritional requirements. Future investigations could therefore seek to quantify the strength of associations between‐sex differences in nutritional requirements and stable isotope values, which could contribute greatly to our understanding of ecological differences between males and females.

## AUTHOR CONTRIBUTIONS

**Joshua T. Bauld:** Conceptualization (equal); data curation (equal); formal analysis (equal); funding acquisition (equal); investigation (equal); methodology (equal); project administration (equal); resources (equal); software (equal); validation (equal); visualization (equal); writing – original draft (equal); writing – review and editing (equal). **Jason Newton:** Conceptualization (equal); data curation (equal); formal analysis (equal); funding acquisition (equal); investigation (equal); methodology (equal); project administration (equal); resources (equal); software (equal); supervision (equal); validation (equal); visualization (equal); writing – original draft (equal); writing – review and editing (equal). **Isabel L. Jones:** Conceptualization (equal); data curation (equal); formal analysis (equal); funding acquisition (equal); investigation (equal); methodology (equal); project administration (equal); resources (equal); software (equal); supervision (equal); validation (equal); visualization (equal); writing – original draft (equal); writing – review and editing (equal). **Katharine A. Abernethy:** Conceptualization (equal); data curation (equal); formal analysis (equal); funding acquisition (equal); investigation (equal); methodology (equal); project administration (equal); resources (equal); software (equal); supervision (equal); validation (equal); visualization (equal); writing – original draft (equal); writing – review and editing (equal). **David Lehmann:** Conceptualization (equal); data curation (equal); formal analysis (equal); funding acquisition (equal); investigation (equal); methodology (equal); project administration (equal); resources (equal); software (equal); supervision (equal); validation (equal); visualization (equal); writing – original draft (equal); writing – review and editing (equal). **Luc F. Bussiére:** Conceptualization (equal); data curation (equal); formal analysis (equal); funding acquisition (equal); investigation (equal); methodology (equal); project administration (equal); resources (equal); software (equal); supervision (equal); validation (equal); visualization (equal); writing – original draft (equal); writing – review and editing (equal).

## CONFLICT OF INTEREST

The authors declare that they have no competing interests.

## Figures and Tables

**FIGURE A1:**
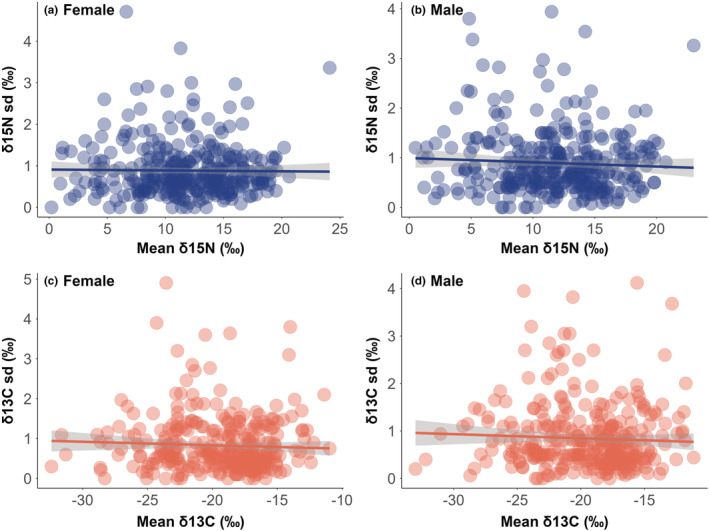
Mean–variance relationships in female nitrogen(a), male nitrogen (b), female carbon (c), and male carbon (d).

**FIGURE A2:**
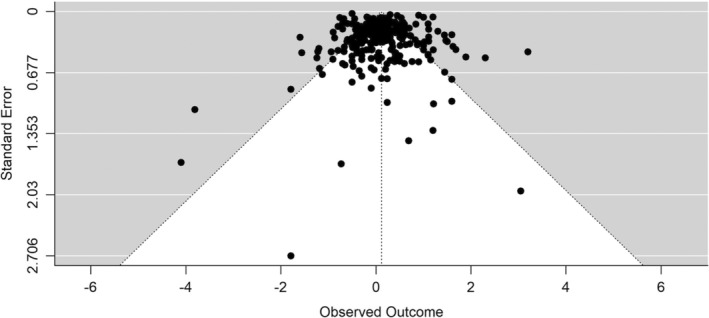
Distribution of published sex differences in nitrogen isotope means.

**FIGURE A3:**
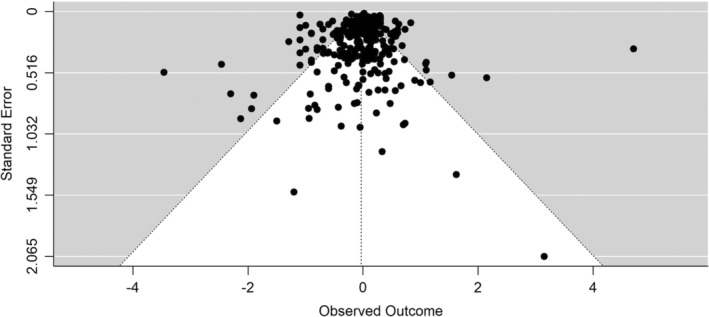
Distribution of published sex differences in nitrogen isotope variation.

**FIGURE A4:**
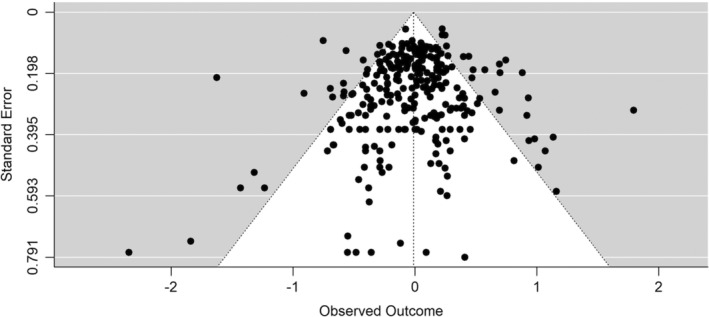
Distribution of published sex differences in carbon isotope means.

**FIGURE A5:**
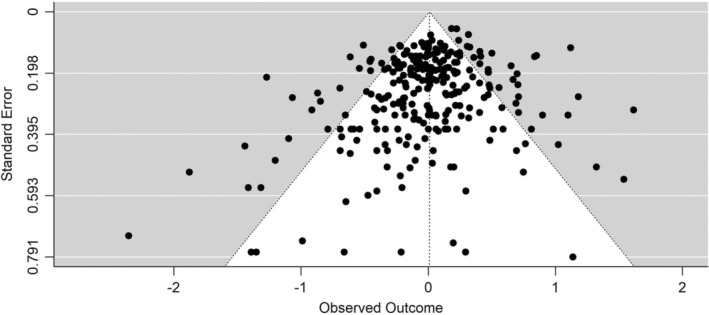
Distribution of published sex differences in carbon isotope variation.

**FIGURE A6:**
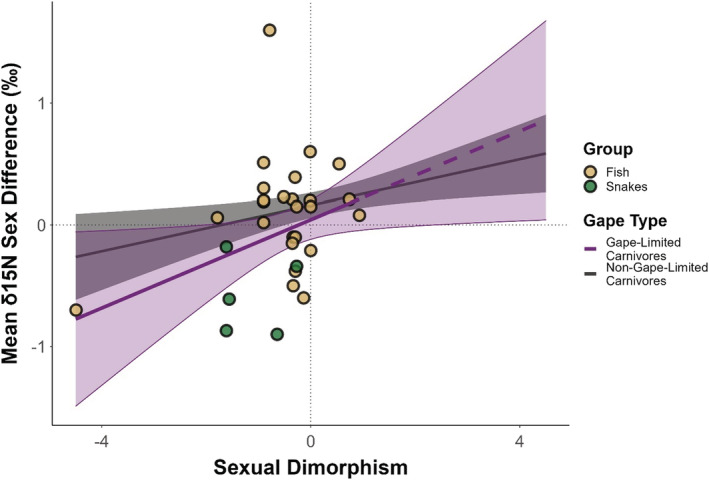
The effect of size dimorphism in gape‐limited predators (purple) and nongape‐limited carnivores (gray) when not controlling for phylogenetic relatedness between species.

## Data Availability

All raw data supporting our analyses, as well as modeling and plotting scripts, will be made available on dryad: https://doi.org/10.5061/dryad.k98sf7m99.

## APPENDIX 1

### MEAN–VARIANCE RELATIONSHIPS

Calculating effect sizes that quantify variation differences between groups, such as sex, may be impacted by mean–variance relationships. These describe an increase in variance with an increase in mean and may adversely influence the outcome of meta‐analyses. We, therefore, plotted the relationship between raw isotopic mean and standard deviation, alongside linear regressions, to investigate the presence of mean–variance relationships in our data. We found no evidence for mean–variance relationships in female nitrogen (Figure A1a), male nitrogen (Figure A1b), female carbon (Figure A1c), or male carbon (Figure A1d).

### PUBLICATION BIAS

#### Publication year

As one possible identifier of publication bias is a reduction of effect sizes through time, we completed meta‐regressions with publication year as the sole predictor variable. We found no effect of publication year on the magnitude of published sex differences in isotopic mean or variation, for either nitrogen or carbon isotopes (Table A1).

**TABLE A1:** Effect of publication year on sex difference in mean and variation, for nitrogen and carbon.

Isotope	Measure	Effect of publication year	Standard error	Confidence interval lower bound	Confidence interval upper bound	*p*
Nitrogen	Mean difference	−0.0010	0.0100	−0.0221	0.0202	.9270
Nitrogen	Variation	−0.0035	0.0066	−0.0164	0.0095	.6010
Carbon	Mean difference	0.0066	0.0081	−0.0094	0.0225	.4195
Carbon	Variation	−0.0094	0.0073	−0.0237	0.0049	.1979

#### Funnel plots

Funnel plots can be used to investigate possible publication bias by illustrating asymmetries in published effect sizes, which would suggest particular results are favorably published. Such biases in published literature would influence the outcome of meta‐analyses, by skewing summary effect size estimates toward the favored outcome. We, therefore, produced funnel plots displaying published effect sizes for sex differences in nitrogen mean (Figure A2), nitrogen variation (Figure A3), carbon mean (Figure A4), and carbon variation (Figure A5). In all four cases, our plots displayed a fairly even distribution in study outcomes, suggesting that publication bias is not prominent in the literature we have examined.

### GAPE LIMITATION: NONPHYLOGENETIC MODELS

When not controlling for phylogeny the effect of size dimorphism in gape‐limited predators was statistically significant and almost double that of nongape‐limited carnivores (Figure A6).

### EFFECT OF STUDY WEIGHTING METHOD

The weight given to individual effect sizes can alter the outcome of meta‐analyses. As our analysis consisted of meta‐regressions, including the random factors “paper number,” “species,” and “phylogeny,” each effect size used as a response in our analysis was weighted accounting for within‐study variance, heterogeneity between studies, species, and phylogenetic relatedness, and covariance between these random factors. Thus, our models assumed differences between studies and species in the true isotopic difference between sexes. However, high heterogeneity, which was present in our data, can mask within‐study variance. It is therefore recommended to also conduct models weighting studies solely by the inverse of within‐study variance, to examine the impact of weighting method on model predictions and, therefore, the conclusions of the meta‐analysis.

Regarding the relationship between‐sex differences in nitrogen mean (trophic level) and size dimorphism, our results were not robust to changing the weighting method, as the confidence interval for the estimated effect of size dimorphism on trophic sex differences overlapped zero (Table A2). Our qualitative conclusion would therefore have changed with the alternate weighting, to state that size dimorphism is unrelated to trophic differences between sexes. However, as this weighting method does not account for between‐study and between‐species heterogeneity in trophic sex differences, we believe it to be inappropriate. The diversity of species investigated by the studies we have meta‐analyzed, and the consequent diversity of our analysis, mean assuming a universal difference in sex differences in trophic level is clearly erroneous. We are therefore skeptical of the conclusion this weighting method produces and are more confident in the original model included in the main text.

When weighting by the inverse of within‐study variance in the model examining the effect of size dimorphism on sex difference in nitrogen variation (Table A3), carbon mean (Table A4), and carbon variation (Table A5), our qualitative conclusions remained the same.

When including dietary class and mean size alongside size dimorphism, as predictors of sex differences in nitrogen mean, the effect of mean size (which was effectively zero) was absent and the effect of size dimorphism was absent in all dietary classes when weighting by the inverse of within study variance (Table A6). Our results in models examining gape‐limited (Table A7) and nongape‐limited carnivores (Table A8) were also not robust to alternate weighting. Finally, the effect of size dimorphism on sex differences in carbon mean sex differences was also statistically nonsignificant when using the alternate weighting method (Table A9). However, for the same reasons outlined above, we are more confident in the original models included in the main text.

**TABLE A2:** Output of fixed effects model examining the effect of size dimorphism on sex differences in nitrogen mean, weighting studies only by the inverse of within‐study variance.

Term	Type	Estimate	Standard error	Statistic	*p*
Intercept	Summary	0.046	0.091	0.51	.61
Size dimorphism	Summary	0.1	0.062	1.63	.1

**TABLE A3:** Output of fixed effects model examining the effect of size dimorphism on sex differences in nitrogen variation, weighting studies only by the inverse of within‐study variance.

Term	Type	Estimate	Standard error	Statistic	*p*
Intercept	Summary	−0.0071	0.041	−0.17	.86
Size dimorphism	Summary	0.039	0.03	1.35	.18

**TABLE A4:** Output of fixed effects model examining the effect of size dimorphism on sex differences in carbon mean, weighting studies only by the inverse of within‐study variance.

Term	Type	Estimate	Standard error	Statistic	*p*
Intercept	Summary	−0.051	0.11	−0.58	.62
Size dimorphism	Summary	0.14	0.08	1.84	.066

**TABLE A5:** Output of fixed effects model examining the effect of size dimorphism on sex differences in carbon variation, weighting studies only by the inverse of within‐study variance.

Term	Type	Estimate	Standard error	Statistic	*p*
Intercept	Summary	0.044	0.039	1.13	.26
Size dimorphism	Summary	0.030	0.033	0.91	.36

**TABLE A6:** Output of fixed effects model examining the effect of size dimorphism, dietary class and species mean size on sex differences in nitrogen mean, weighting studies only by the inverse of within‐study variance.

Term	Type	Estimate	Standard error	Statistic	*p*
Intercept	Summary	0.21	0.076	2.76	.0059
Size dimorphism	Summary	0.059	0.06	0.99	.32
Herbivore	Summary	−0.71	0.3	−2.38	.017
Omnivore	Summary	−0.53	0.28	−1.89	.059
Mean size	Summary	0.00000097	0.0000027	0.37	.71
SSD: Herbivore	Summary	0.65	0.55	1.19	.23
SSD: Omnivore	Summary	0.38	0.27	1.43	.15

**TABLE A7:** Output of fixed effects model examining the effect of size dimorphism on sex differences in nitrogen mean, in gape‐limited carnivores, weighting studies only by the inverse of within‐study variance.

Term	Type	Estimate	Standard error	Statistic	*p*
Intercept	Summary	0.11	0.2	0.55	.59
Size dimorphism	Summary	0.11	0.16	0.73	.47

**TABLE A8:** Output of fixed effects model examining the effect of size dimorphism on sex differences in nitrogen mean, in nongape‐limited carnivores, weighting studies only by the inverse of within‐study variance.

Term	Type	Estimate	Standard error	Statistic	*p*
Intercept	Summary	0.23	0.087	2.6	.0094
Size dimorphism	Summary	0.054	0.066	0.82	.41

**TABLE A9:** Output of fixed effects model examining the effect of size dimorphism on sex differences in nitrogen mean, in nongape‐limited carnivores, weighting studies only by the inverse of within‐study variance.

Term	Type	Estimate	Standard error	Statistic	*p*
Intercept	Summary	0.030	0.07	0.43	.67
Size dimorphism	Summary	0.087	0.074	1.14	.26
Herbivore	Summary	−0.89	0.3	−3.01	.0026
Omnivore	Summary	−0.086	0.28	−0.31	.76
Size dimorphism: Herbivore	Summary	1.3	0.47	2.8	.0051
Size dimorphism: Omnivore	Summary	0.28	0.21	1.33	.18

### HIGH LEVERAGE DATA POINTS

The outcome of meta‐analyses may also be adversely impacted by outliers/high leverage data points that skew model estimates. We therefore used Cook's leave‐one‐out analysis to identify high leverage data points that may have an unduly large effect on our models. Several approaches are possible for identifying data points as high leverage based on Cook's scores, and we chose to assign those with a Cook's score over three times the mean score, for data points in a given model, as potentially high leverage. We found this approach to be the most conservative, by identifying the highest number of points as possibly high leverage. We then removed these data from the models for which they may be high leverage and re‐ran each model.

In models examining only the effect of size dimorphism on sex differences in nitrogen mean (Table A10), nitrogen variation (Table A11), carbon mean (Table A12), and carbon variation (Table A13), removing high leverage data points did alter estimated effect sizes but did not change the qualitative conclusions we could draw from the models. When including dietary class and species mean size as predictors, alongside size dimorphism, the effect sizes changed (Table A14), but our qualitative conclusions did not. In models analyzing the impact of size dimorphism on sex differences in nitrogen mean in gape‐limited (Table A15) and nongape‐limited carnivores (Table A16), removing high leverage data points also did not change our qualitative conclusions. Finally, when removing high leverage data points from the model examining the effect of size dimorphism and diet on sex differences in carbon mean sex differences, the effect of size dimorphism in herbivores was no longer significant (Table A17). This is likely because of the small sample size of herbivores in our data set, rather than an indication that any points should be removed.

**TABLE A10:** Output of meta‐regression model examining the effect of size dimorphism on sex differences in nitrogen mean, with high leverage data points removed.

Term	Type	Estimate	Standard error	Statistic	*p*
Intercept	Summary	0.076	0.039	1.95	.051
Size dimorphism	Summary	0.2	0.053	3.57	.00036

**TABLE A11:** Output of meta‐regression model examining the effect of size dimorphism on sex differences in nitrogen variation, with high leverage data points removed.

Term	Type	Estimate	Standard error	Statistic	*p*
Intercept	Summary	−0.012	0.029	−0.43	.67
Size dimorphism	Summary	−0.0048	0.033	−0.15	.88

**TABLE A12:** Output of meta‐regression model examining the effect of size dimorphism on sex differences in carbon mean, with high leverage data points removed.

Term	Type	Estimate	Standard error	Statistic	*p*
Intercept	Summary	0.022	0.03	0.74	.46
Size dimorphism	Summary	0.063	0.039	1.63	.1

**TABLE A13:** Output of meta‐regression model examining the effect of size dimorphism on sex differences in carbon variation, with high leverage data points removed.

Term	Type	Estimate	Standard error	Statistic	*p*
Intercept	Summary	−0.0027	0.027	−0.1	.92
Size dimorphism	Summary	0.031	0.028	1.11	.27

**TABLE A14:** Output of meta‐regression model examining the effect of size dimorphism and dietary class and species mean size on sex differences in nitrogen mean, with high leverage data points removed.

Term	Type	Estimate	Standard error	Statistic	*p*
Intercept	Summary	0.1	0.044	2.35	.019
Size dimorphism	Summary	0.18	0.051	3.45	.00055
Herbivore	Summary	0.032	0.46	0.069	.95
Omnivore	Summary	−0.097	0.11	−0.9	.37
Species mean size	Summary	0.0000064	0.0000025	2.62	.0088
Size dimorphism: Herbivore	Summary	0.15	0.64	0.24	.81
Size dimorphism: Omnivore	Summary	0.44	0.23	1.93	.054

**TABLE A15:** Output of meta‐regression model examining the effect of size dimorphism on sex differences in nitrogen mean, in gape‐limited carnivores, with high leverage data points removed.

Term	Type	Estimate	Standard error	Statistic	*p*
Intercept	Summary	0.066	0.15	0.45	.66
Size dimorphism	Summary	0.025	0.096	0.26	.79

**TABLE A16:** Output of meta‐regression model examining the effect of size dimorphism on sex differences in nitrogen mean, in non‐gape‐limited carnivores, with high leverage data points removed.

Term	Type	Estimate	Standard error	Statistic	*p*
Intercept	Summary	0.12	0.054	2.19	.029
Size dimorphism	Summary	0.19	0.077	2.53	.011

**TABLE A17:** Output of meta‐regression model examining the effect of size dimorphism and dietary class on sex differences in carbon mean, with high leverage data points removed.

Term	Type	Estimate	Standard error	Statistic	*p*
Intercept	Summary	0.014	0.036	0.38	.7
Size dimorphism	Summary	0.028	0.043	0.66	.51
Herbivore	Summary	0.45	0.49	0.91	.36
Omnivore	Summary	−0.11	0.086	−1.28	.2
Size dimorphism: Herbivore	Summary	−0.41	0.9	−0.46	.65
Size dimorphism: Omnivore	Summary	0.29	0.19	1.51	.13
